# Short Communications: Monitoring an average temperature in an automatic density assembly

**DOI:** 10.1155/S1463924681000102

**Published:** 1981

**Authors:** M. E. B. Brown, R. G. Lidzey

**Affiliations:** 1 The Laboratory of the Government Chemist Cornwall House Stamford Street London SE1 9NQ UK; 2 Plasrna-Therm Ltd. 6 Station Rd. Penge London SE20 UK


capital cost is higher than some of its competitors it has the advantage of carrying out kinetic analyses in the KIN mode with monitoring by the operator for linearity within limits specified and hence producing a result of higher quality.

ACKNOWLEDGEMENTS
The authors wish to thank Boehringer Corp. Ltd. for the opportunity to carry out this evaluation and for supplying many of the reagents, and especially Mr Michael Clegg for his help and cooperation.

REFERENCES
[1] Trinder, P., *Journal of Clincial Pathology*, 1969, **22**, 246
[2] Moorehead, W. R., and Biggs, H. G., *Clinical Chemistry*, 1974, **20**, 1458
[3] Stromme, J. H., and Eldjarn, L., *Scandinavian Journal of Clinical Laboratory Investigation*, 1974, **33**, 291
[4] Searcy, R. L., Simms, N. M., Foreman, J. A. and Bergquist, L. M., *Clinica Chimica Acta*, 1965, **12**, 170
[5] "Electrical Safety Code for Hospital Laboratory Equipment, 1977" HMSO, London.

### Monitoring an average temperature in an automatic density assembly

**M.E.B. Brown* and R.G. Lidzey**
*The Laboratory of the Government Chemist, Cornwall House, Stamford Street, London, SE1 9NQ, UK.*

In the continuous measurement of temperature in a temperature-dependent system it may be inconvenient or impossible to make the measurement at the site where the variation is most important. This may, for example, occur in a water jacketed temperature controlled arrangement where there is difficulty in insulating sensor leads or fitting a mercury in glass thermometer due to the proximity of other equipment. In such cases it may be acceptable to take the average value at two or more other local sites, and in the example quoted an average temperature derived from sensors located in the inlet and exit water streams may be just as satisfactory.

The determination of strength of alcohol solutions by density requires precise temperature control. In an automated assembly [1] using a commercially built densimeter, the Anton Paar DMA55 (available from Standon Redcroft, UK) it was found necessary to have a continuous display of the density cell temperature. A variation of 0.01°C will introduce an error of 1 in $10^5$ in the density or 0.01% v/v in the apparent alcohol strength.

Thermistors were fitted into limbs of the thermostated water supply external to the instrument for the reasons stated above. Although a small diameter thermometer pocket is fitted on the cell, a thermistor fitted here is found to give misleading temperature indications and the present arrangement is preferred. Densities are measured at a nominal temperature of 20°C and a digital display of the temperature is given as a positive or negative departure from this value with a sensitivity of $10^{-3}$°C and a range of ± 2°C.

**Present address: Plasma-Therm Ltd., 6 Station Rd., Penge, London SE20, UK.*

### Circuit details

The two thermistors are connected in series in a bridge circuit forming one leg of the bridge (Figure 1). The remaining legs contain fixed resistors of the same total value. A voltage reference stabilised by two zener diodes supplies the bridge. Output of the bridge is via the 5.6 volt zener diode selected for its low temperature coefficient. The two 50 kohm resistors between the bridge and the first operational amplifier reduce the effect that the changing impedance of the thermistors have on the gain of the amplifier. A 0.35 volt potential drop across the forward biased signal diode provides a voltage offset to the second operational amplifier. The voltage is adjustable by a 5 turn ten k ohm potentiometer. The gain of the second operational amplifier is adjustable so that a change of ± 1.999°C in the mean temperature is displayed as ± 1.999 on the panel meter. The components are selected for stability, the resistors are TR5 type, the preset 5 k ohm twenty turn potentiometer is a wire wound precision type by Bourns Trimpot (Hadford House, 17/27 High Street, Hounslow, Middlesex TW3 ITE UK) and the thermistors are YS1 precision type 44031. (Sasco Ltd, PO Box 2000, Crawley, Sussex, RH10 2RV UK). The operational amplifiers, OP07 by Precision Monolithics, supplied by Bourns address have an ultra low offset voltage and current drift. A mains operated power unit by Gresham Lion (Gresham House, Twickenham Road, Feltham, Middlesex TW13 6HA UK) supplies ± 15VDC. A convenient check on the circuit function can be made by switching out the thermistors and connecting in a resistance equal to the combined thermistor values at 20°C.

The unit was calibrated by immersing both thermistors in a bath and adjusting the gain of the second operational amplifier over the ±2°C range with the 5 kilohm potentiometer and then setting the panel meter display to 0.000 for 20°C with the offset control. The sensitivity is 0.001°C with a 24 hour drift not exceeding ±0.005°C. When both temperatures are within ± 0.5°C of 20°C, the maximum error due to the non-linear characteristics of the thermistors is 0.01°C.

REFERENCES
[1] Submitted for publication Journal of Automatic Chemistry.

